# Dr. Reid on Puerperal Insanity

**Published:** 1848-01-01

**Authors:** James Reid

**Affiliations:** PHYSICIAN TO THE GENERAL LYING-IN HOSPITAL, ETC.


### ON THE CAUSES, SYMPTOMS, AND TREATMENT OF PUERPERAL INSANITY.

BY JAMES REID, M.D.

PHYSICIAN TO THE GENERAL LYING-IN HOSPITAL, ETC.

The term, puerperal insanity, is not only understood to imply aberration of the mind, or derangement of the cerebral functions in the puerperal state itself, but to include those attacks which occur sometimes during the period of gestation, as well as those which we more frequently meet with some months after partrition, whilst the patient is suckling her infant.

From the period of conception, during the whole term of gestation, and up to the termination of suckling, there is an amount of nervous irritability and excitement in the system, which strongly predisposes to any cerebral affection; but the two principal epochs at which this excitability becomes the most dangerous are *immediately after parturition*, and at a later period, when the system is exhausted by a too long-continued application of the infants to the breast. We find cases recorded in which cerebral disorder has commenced immediately after conception, and which has ceased at the period of quickening: others, again, have persisted throughout the whole term of gestation, but terminated on delivery taking place; whilst more rare cases have still continued until lactation was relinquished.

The whole cerebro-spinal system is much excited during pregnancy, and more especially in the puerperal state; the senses are often extremely vivid, and the slightest impressions agitate the mind, which is thus ready to receive any false impressions which may be brought out by a sudden shock, or unexpected and exciting cause: thus the powerful influence of fright, surprise, or other strong emotions, in this condition of the nervous system acting on a mind already disposed to mania by some hereditary influence.

It fortunately happens, that it is not often we have the opportunity of observing in private practice this distressing malady; even in the public lying-in establishments of this country, the complaint is much more rarely noticed than it would appear to be in the hospitals of France; and considering that physical and moral causes of disturbance are so rife during pregnancy and labour, it is surprising that the proportion of such cases should be so small.

There are very few works devoted solely to this form of the disease in our own language, but there are several excellent descriptions of it in some of the treatises on mental alienation in general: perhaps the best of these is one by the late Dr. Gooch, in a chapter "On the Disorders of the Mind in Lying-in Women," contained in his valuable work "On the most important Diseases peculiar to Women," 1829, written in that clear practical style for which he was always remarkable.

The chapter, "De l'Aliénation mentale des nouvelles Accouchées et des Nourrices" in the work on insanity of Esquirol, is well worth an attentive perusal by those who are interested in the subject.

*Any serious disturbance in the uterine functions* speedily affects the brain; and to those conversant with the peculiar diseases of females, numerous instances of corresponding cerebral complaints following such disturbance, will at once be in their remembrance. The uterus and ovaries seem to exert the strongest sympathetic action upon this distant organ; and should even the usual monthly function be irregular, impeded, or altogether checked for a time, the cerebral tissues appear to share immediately in the disorder, as evidenced by headaches, giddiness, depression of spirits, or sometimes by great excitability, the whole system at length becoming likewise affected, and requiring judicious medical treatment. In some cases, this state alone has even been sufficient to cause an attack of insanity.

One epoch of life, at which females are peculiarly liable to this complaint, is that which is usually denominated *the critical period,* when the monthly function ceases altogether. In the Hanwell Reports for the last eight years, there are eight cases given as proceeding from this cause. With those who have been previously affected with mental aberration, great care and preventive measures will be especially required to guard against a return at this age.

In younger patients, suffering from mania and melancholia, I have known more than one instance of recovery following the re-appearance of the catamenial discharge, which had heretofore been suspended. It may, however, be a question, is this cause or effect? Monsieur Voisin, in a prize essay on the question, 'Is irregular menstruation to be regarded as one of the most frequent physical causes of mental alienation?" &c., maintains, that it is the *result* of the affection of the brain, and that whilst this remains in its active state, no return of the menstrual function takes place. Now this is a statement certainly not borne out by facts; for though the impeded or irregular function very frequently accompanies attacks of insanity in females, still I have known several cases in which menstruation went on regularly during such illness. In some others it has been more profuse than usual. A case occurred during the last month, in which a young female, who was received into the infirmary of St. Giles, labouring under an attack of mania, was suffering at the same time from menorrhagia. In the case of a lady aged 36, whom I visited in consultation with Dr. Allan of Islington, on more than one occasion, attacks of insanity have occurred, frequently attended with most severe pain, principally seated in the left brow and parietal region, with a sensation as if some one were constantly hammering at the parts; the pulse was not much affected, ranging from 80 to 85 in the minute; tinnitus aurium—insomnia—clean tongue: *the catamenial discharge is most profuse at each period,* lasting from ten to fourteen days, and, on its disappearance, it is succeeded in the intervals by a leucorrheal discharge. The attacks are of so serious a nature that great care is required to prevent self-injury, or violence towards her child, which she has threatened whilst thus affected.

An instance is related by Mons. Marc (Consultation Médico-légale) of a young female aged twenty-seven, whose bodily functions were perfect, but still at each approach of the catamenial period, a degree of excitability appeared, which became dangerous to those about her, she at these times being attacked by a desire to stab with a knife. Having on one occasion carried this threat into execution, she was consigned to a lunatic asylum.

In the case of a young lady, for which I was consulted about two years since, caused by her lover deserting her, and marrying her sister, although there was a very irritable state of the uterus, yet the monthly function occurred regularly throughout a long period.

Suppression of the menses, however, will alone act as the exciting cause of insanity in some cases, in young delicate females, and especially when combined with great irritability of the uterine organs. Hysteria is often an effect of this, and sometimes epilepsy.*

In the Hanwell Reports, out of 703 female cases of insanity, ten are noted as arising from *suppressed menstruation,* and nine from uterine excitement.

Of 361 cases in the Salpêtrière, arising from physical causes, there were fifty-five originating from "irregularity of menstruation," and twenty-seven occurring at the critical period.

Dr. Conolly has well remarked—"The state of the uterus in the unmarried, and in women recently confined, is often the cause of mental disturbance of a most formidable aspect; and if the cause is overlooked, the patient will generally be treated unsuccessfully." (Indications of Insanity, 1830.)

### HYSTERICAL MANIA.

There is a species of aberration of the intellect occurring in young females, which evidently is complicated with hysteria; the peace of a whole family is for the time totally destroyed by the whims, extravagancies, perverseness of temper, and violence of the patient: some of these cases border closely on insanity, but others, when protracted beyond a certain period, gradually merge either into mania or melancholia, and it then becomes absolutely requisite to remove them from the society of their friends, and to place them under the charge of some practitioner who is experienced in the treatment of such cases.

They are often termed nervous disorders, but these, unless closely watched, and carefully treated, are prone to degenerate into more fixed and permanent affections of the brain. The following may be taken as one of the above description of cases. Miss ——, aged fifteen, of tall,

* The suppression of the catamenial discharge is not unfrequently attended by costiveness and torpidity of the liver; in such cases, alteratives, emmenagogues, and the application of a few leeches to the feet, or to the vulva, will be the most likely means of restoring the healthy secretions, and of relieving the cerebral irritability.

robust frame, menstruating irregularly, had laboured under various forms of hysteria for a short time past, and complained of violent pain in the uterine region, tympanitic distension of the abdomen, torpid bowels, headache, sense of suffocation, and of such a constant feeling of restlessness, that it was difficult to keep her in the room: she gradually became exceedingly violent—and her long-continued fits of screaming often alarmed the whole neighbourhood: on two occasions it was found requisite to put her under restraint. The medical attendant was nearly worn out by the constant attendances required day and night to introduce the catheter, &c., but he almost invariably found that the bladder contained little or no urine, although the attendants asserted positively that none had been passed naturally. When I saw her I felt convinced that such must have been the case; her pulse was natural, her strength good, and there were no symptoms which must have existed had the assertion been true: the greatest distress was occasioned in the family by the state of the patient during two months of violence, as they were unwilling that she should be separated from them; at the end of this period a sort of stupor came on, and continued for a week, on recovering from which, the catamenial function continued regularly, symptoms of mental recovery became stronger, and for some years past there has not been the slightest appearance of a similar attack. I should add, that on very close watching, it was discovered that the patient had regularly passed urine, but had contrived to avoid the notice of the nurse for some days at such times.

### INSANITY DURING PREGNANCY.

We have already seen that deranged uterine function, especially when accompanied by great local excitability, is sufficient of itself in some cases to cause insanity; we are therefore prepared to understand that in such a disturbed and excited state of the uterine system, as is the result of pregnancy, and more especially of the parturient period, coupled too, as they are with great mental excitement and physical shock to the whole frame, there must be a still greater liability to insanity present. In some cases of pregnancy, a curious change relative to the cerebral functions has been noticed as occurring; viz., one faculty being suspended whilst another is substituted in its place. Thus in Goubelli's case, a female during the term of her gestation lost her *memory*, which at other times was extremely tenacious, and in its place a *sound judgment*, which she was otherwise not remarkable for, was substituted: after her delivery, the judgment again became obtuse, whilst her memory returned! It is well known to all those who are conversant with the treatment of pregnant females, that this state is not unfrequently attended by a remarkable change in the temper and habits. Conception itself is sometimes speedily notified by an unusual degree of mental and bodily excitement, the imagination becomes exalted, impressions are more lively, and there is an abnormal condition of the whole nervous system: unusual feelings and desires (especially in articles of diet) are noticed, as well as eccentricities and caprices, and sometimes a latent talent for poetry, music, or conversation, is called forth during this period, which had not previously been known to exist.

Amongst these eccentricities, a great relish for every article which could be secretly purloined from others has been noticed by Baudelocque and several other writers : some other equally awkward propensities have been recorded also. Now, all due allowance is generally made for innocent whims and caprices during the term of gestation, for the longings, &c. whilst they are kept within bounds, as not being altogether unnatural, but when any one or more of these eccentricities assume a magnified importance in the brain of the patient, and a power which the mind is unable properly to control, they may at length lead to sudden impulses, which, if not dangerous, still may be of that serious character, as to require much watching and management on the part of her friends.

Dr. Forbes Winslow has lately drawn the attention of the profession more forcibly to the "incubation of insanity," in an interesting paper. Some of the foregoing symptoms form part of those noticed as precursory ones during this incubation: it is sometimes detected by various physical, and especially by moral symptoms—headache, malaise, irritability, restlessness of manner, uneven spirits, love of change, want of sleep or frightful dreams, remarkable modification of character, either the usual one exaggerated, or of a different nature. Anxiety, distrust, suspicion, melancholy, fear of the future, may separately or conjointly assume a prominent importance, and at length a slight hallucination may even appear in relation to one particular subject; such, for instance, as that a particular friend has offered some imaginary insult or annoyance, and this feeling, which at first is looked upon as a mistaken sensitiveness only, is gradually recognised as the effect of morbid imagination, and a more full explanation is perhaps speedily afforded by a sudden explosion of maniacal violence, or by a settled melancholy.

The caprices or casual dislikes soon ripen into morbid fantasies and inveterate antipathies, until some one idea gradually appears to rise out of all the other phantasmagoric illusions, and to fix itself indelibly on the mind, assuming an ideal importance, and a total control over it. It is haunted constantly by this fixed idea (monomania), and cannot abstract itself from it, as it seems to mingle with every occupation and pursuit.

The cases of insanity which arise *during* pregnancy are much smaller in number than those which *follow* it, and the majority of the former terminate with the occurrence of labour. They are more apt to occur in the later months of pregnancy than in the earlier ones; although Esquirol mentions the case of a female who was attacked, on two different occasions, by insanity immediately after conception, each attack lasting for a period of fifteen days. A female was received into the Bethnal-green Asylum who had been attacked by melancholia immediately after quickening, with a strong desire to destroy herself and her three children; it continued during the remainder of pregnancy, and became worse after delivery.

In another case of the same asylum, strangeness of conduct was observed a month before her confinement, perfect incoherence and great depression continuing afterwards.

This description of case, however, is not, I believe, frequently to be met with in public asylums.

It occasionally has happened that pregnancy, taking place in females who were insane already, has completely restored them during the period of gestation.

Insanity is sometimes produced during pregnancy by sudden fright. Boivin relates the case of a young woman, in her sixth month of pregnancy, who from this cause became idiotic, lost her voice, and was so hideous in appearance, that the other patients in the asylum to which she was removed called her "*the vampire.*" Notwithstanding this extreme state, gestation progressed favourably, and by judicious treatment she recovered in three weeks, and went on to her full time, without any return of the complaint.

It is sometimes the case, that although the female herself may escape insanity from severe mental shocks, still her offspring unfortunately does not, and congenital insanity is its lot. Three such cases are mentioned in the Hanwell reports. One case has fallen under my notice, in which the mother was suddenly terrified by a fatal accident occurring to one of her children, during the time she was pregnant with another; and the latter, now of adult age, though not afflicted by insanity, is at all times of so extremely nervous a temperament as to be unfitted for any of the active duties of life. Females who, from hereditary or other causes, have a peculiar predisposition to insanity, appear to be much more susceptible, as to its attacks, during pregnancy, or soon after its termination.

### INSANITY OCCURRING AFTER LABOUR.

There are two forms of delirium, occurring during labour and after it, which have by some authors been included under the denomination of puerperal insanity, but, I think, without reason. The one is that passing delirium, the result of intense temporary pain and excitement in labour, or which attends the febrile action so common about the fourth or fifth day after this, when the secretion of milk is fully established; the *other* is the delirium which is not unfrequent in fatal cases of puerperal fever, and in which, from ample opportunities of observation in the lying-in wards and fever wards of St. Giles's Infirmary for some years past, I have never been able to detect any difference in their nature from that so common in typhus. I do not allude to the low, muttering delirium, but to those sudden attacks of cerebral excitement which may take place in either of these two species of fever, so as to require restraint for hours together, and even for days in some cases. The excitement in either fever is not always one of violence, however; for I have seen in both forms the greatest gaiety and vivacity, as evidenced by singing, joking, and laughing, a few hours previous to death, but this more especially in puerperal fever. In both diseases some metastasis appears to occur, and most probably by means of the alteration which takes place in the condition of the blood. There is, however, one important difference between the two diseases when this symptom appears, as to prognosis; it is generally a fatal one in puerperal fever, but not so in common typhus. In the delirium and temporary mania both of typhus and puerperal fever, there is not *always* total derangement of all the intellectual faculties; the patient can frequently be roused out of this state, by speaking in a loud tone, and for a short time is able to retain the power of answering questions, but soon subsides into the rambling incoherence of ideas.

*Puerperal insanity* seldom appears sooner than the third day after labour (about the time when the secretion of milk is fully established), or later than the fourteenth day, when the discontinuance of the lochial discharge usually occurs. The shock and excitement, both physical and mental, which attend the act of parturition, leave the cerebro-spinal system in a morbidly susceptible state, attended by great exhaustion of the vis vitæ.

Now, we well know that exhaustion and irritability combined, when following great excitement, long continued and kept up by the free use of stimulants, will produce precisely a similar effect upon the brain, as evidenced in cases of *delirium tremens;* so that there is a strong analogy between the two diseases, although arising from different causes. This subject more especially requires our attention in considering the *treatment* of puerperal mania.

The attack is often preceded by several premonitory symptoms, or may suddenly take place without them. In the former case, some eccentricity of manner is not unfrequently noticed, even before the occurrence of labour,—such as great susceptibility, unusual display of suspicion as to those around, an irritable oddity of manner, &c. In the case of a lady whom I attended some time since, she had surprised her friends on more than one occasion, during the latter period of her pregnancy, by taking offence suddenly at some of them for looking rudely at her.

In other cases, there is a sudden excitability of the sensorial functions observed a few days after parturition; an unusual flow of spirits, great volubility of language, a crowding in of thoughts and images upon the mind, so that the patient confesses that she is incapable of thinking or reasoning calmly; a little incoherence is then observed, uncontrollable mirth and laughter at trifles, restlessness, inability to sleep or even to compose herself, the thoughts wander and are embarrassed, and the brain appears to gradually lose its power of control: the insomnia soon becomes a prominent symptom, lasting for several nights together, and in some cases, anodynes appear to exert no beneficial influence, but rather to increase the irritability and watchfulness. At length a sudden paroxysm of maniacal violence explodes, or the patient subsides into a taciturn state of melancholy, from which it is impossible to arouse her.

The patient is sometimes able at the commencement of the complaint to recognise the occasional aberrations of her mind, or the latter appears to be sensible of its errors, and strives to avoid or subdue them, but in vain.

As the affection of the brain progresses, all the former symptoms become, as it were, exaggerated; the talking becomes almost incessant, and generally on one particular subject, such as imaginary wrongs done to her by her dearest friends; a total negligence of, and often strong aversion to her child and husband are evinced; explosions of anger occur, with vociferations and violent gesticulations; and although the patient may have been remarkable previously for her correct, modest demeanour, and attention to her religious duties, most awful oaths and imprecations are now uttered, and language used which astonishes her friends. The eye is wandering and unsteady, the hearing most acute, and the whispered observations of the attendants distinctly heard and commented on, but vaguely and incoherently: there is often, too, a somewhat acute though incorrect mode of arguing on the false idea which may predominate in her mind. The patient's indignation is sometimes directed against some imaginary person, or roused by some ideal obstacle raised to her wishes. A restlessness and anxiety to go to some other place, without giving any reason, or, in fact, knowing why. Illusions succeed each other in quick succession, or settle into one fixed *monomania*. The most usual one which I have observed in females, is that connected with religion, such as the idea of having committed some unpardonable sin, &c. Fear of poison in the food haunts the mind, or, as in one unfortunate case under my care, the idea that murder was constantly going on around her, and that every person whom she saw, and all the food brought to her, was covered with blood, for a long time prevented her taking any nourishment. A frequent delusion is that of hearing voices speaking to her; and in some unfortunate cases the supposed directions of such hidden powers, as to the commission of suicide, have been too implicitly obeyed. This *suicidal tendency* is not uncommon, especially in the cases of melancholia; and it is important always to recollect the fact in the treatment of such patients. In 111 cases of puerperal insanity at Bethlehem Hospital, thirty-two were affected by it.

As in other forms of mania, whilst the mother is urged on by some unaccountable impulse to commit violence on herself or on her offspring, she is at the same time impressed with horror and aversion at the crime. The infant is usually the object of it in puerperal insanity; an impulse to destroy, haunts the mind continually, and struggles with maternal tenderness, which as strongly checks the act. The sufferer, in some cases, implores that the infant may be removed from her, lest she should altogether lose her self-control, and is heard praying to Heaven to prevent her from yielding to the temptation. In one of the lying-in hospitals of London, not long since, the mother did actually destroy her child during one of her paroxysms.

In some cases, the patient suddenly exhibits the maniacal symptoms, without any antecedent ones. On awaking out of an apparently quiet slumber, she shrieks out, and exclaims that her infant is dead, or that it has been taken from her. Nothing can persuade her to the contrary, not even the sight of the child itself in some cases.

The *physical symptoms* are not invariably the same; the secretion of milk is generally, but not always diminished; headache, and a peculiar feeling of discomfort and pain in the head, are described by the patient in her lucid intervals; the bowels are constipated, the tongue white and coated, skin sometimes hot, and pulse rather quick, but weak.

As in all other puerperal complaints, the *quick pulse* is the symptom which causes most anxiety to the obstetric practitioner, as experience has taught us, that this is a symptom which denotes more accurately the approach of danger than any other sign, and it may be laid down as an axiom, "that whilst the pulse remains above 100 in the minute, in any case after labour, it must always be regarded as a sign of approaching evil."

Dr. William Hunter and Gooch have particularly adverted to the state of the pulse, as indicative of a dangerous form, if it is *rapid;* the other and milder form of the complaint is accompanied by a very moderate disturbance of the circulation. The latter cases are the most numerous, and likely to be cured; the former generally die.

Febrile symptoms are, then, not necessarily present in puerperal mania. The digestive organs are more frequently in fault; but this would naturally be expected when there is great excitability of brain, and is likely effect and not cause. Dark offensive dejections are often passed; the breath is likewise offensive, and the lips and teeth are sometimes coated with sordes.

The patient appears to be little affected by cold, and it not unfrequently happens that she endeavours to divest herself of her clothing, and to walk about uncovered.

The constant state of excitement soon affects the general health. Emaciation is a frequent effect; a pallid, jaundiced look, with sunken eyes, is observed, and great prostration of strength succeeds, if the attack is long continued.

*Period of attack.*—Of ninety-two cases at Salpêtrière, sixteen commenced before the fourth day after accouchement, twenty-one before the fifteenth, seventeen before the sixtieth, nineteen in the twelve months after, and nineteen immediately after weaning.*

M. Esquirol draws the conclusions—

1. That insanity is more likely to occur after labour, than at a later period.
2. That the danger of an attack diminishes as the period is more distant from the labour.

*Hereditary tendency* is one cause of puerperal insanity, and in many cases of the complaint we feel assured that it would not have occurred at all, were there not already in the patients a peculiar pre-aptitude for its aggression.

In the 111 cases in Bethlehem, there were forty-five in whom an hereditary tendency was ascertained.†

There is one form of madness, or raving, which sometimes, though very rarely, attacks puerperal patients, so different in its cause from common mania, and requiring such totally different treatment, that it deserves especial notice.

I allude to PHRENITIS, or inflammatory affection of the brain and its membranes.

This form generally appears at an earlier period after labour than mania, and is a most dangerous malady. As in other serious puerperal diseases, the pulse is hard and very quick from the commencement; it

* Esquirol, p. 234.

† For an excellent abstract of the cases occurring in the Bethlehem Lunatic Hospital, during the last five years, I am indebted to the kindness of my friend, Dr. John Webster.

often sets in with a rigor, and a distressing headache, vertigo, pyrexia, great heat of scalp, dry skin, scanty secretion of urine, throbbing in the temples, flushed cheeks, ringing in the ears, intolerance of light, total want of sleep; the vessels of the conjunctiva are injected, painfully acute hearing, hurried manner, confusion of thought, the ideas are incoherent, and the patient cannot pursue any chain of reasoning, even on false data; she becomes unconscious often of surrounding objects, and it is succeeded at length by furious mania. Here, then, is inflammatory action going on, with strong determination of blood to the cerebral vessels, which must be speedily subdued, or no chance exists as to the safety of the patient; if not so, the usual effects of inflammation soon supervene—viz., effusion on the brain, paralysis, coma, convulsions, and death.

The fatal termination often takes place by the fourth day, and almost always within a week from the commencement of the attack, and is heralded by the shrunk features, cold clammy sweats, glazed eye, cold extremities, and laborious respiration. If there is to be a favourable termination, it is notified by a speedy decline of the inflammatory symptoms, and the delirium gradually becomes milder, and is at length subdued.

Collins mentions a case which occurred in the Dublin Lying-in Hospital, twenty-eight hours after delivery, and notwithstanding most active treatment, she died comatose fifty-five hours after the commencement of the attack. On a post-mortem examination, the serous covering of the brain exhibited, in some places, an opaque and thickened appearance, the ventricles being distended, and containing fluid to the amount of four tablespoonfuls.—(Practical Treatise on Midwifery.)

One principal diagnostic mark will be, the appearance of febrile and inflammatory cerebral symptoms, *alone at first*, which are *followed* by mental aberration.

W. Hunter attributed his *fatal* cases with quick pulse to paraphrenitis, but Gooch was of opinion that they rather depended on "exhausting excitation of the nervous system."

### INSANITY FROM UNDUE OR PROTRACTED LACTATION.

If there is any doubt in the minds of practitioners as to whether *mania* depends upon inflammatory congestion, or mere excitability, there is, at least, little in *this form* of the complaint, which evidently owes its origin to the exhaustion of sensorial power. In all cases we find an adynamic state of the system, or great depression of the vital powers.

Several premonitory symptoms serve to usher in this malady in the majority of cases, but not invariably, as in exceptional cases the attack comes on without any previous warning. Anxiety of mind without apparent cause, sleeplessness, alteration of temper and habits, great depression of spirits, peevishness, listlessness, loss of memory, giddiness, headache, and confusion of intellect, generally herald in the approach of this form of insanity, and the patient herself expresses a feeling that something is going wrong as to her brain. Patients thus attacked are observed to be generally emaciated, with an anæmic, pale appearance, to have lost their appetite, complaining of a dragging, sinking sensation from the epigastrium to the spine always after suckling the infant, and showing every symptom of great exhaustion and debility. It is, in fact, a state of excitability combined with great loss of power.*

There are many cases to be met with in private practice in which mothers who have injudiciously persisted in suckling their infants, when it was evident that their strength was becoming materially impaired, as proved by loss of flesh, weak pulse, loss of appetite, and languor, have at length been affected by a distressing lowness of spirits, and dejection of mind, with some undefined fear as to themselves or their infants. Now these, I have no doubt, would speedily have merged into melancholia, had not decisive steps been taken to counteract its progress, by insisting upon weaning, by the use of tonics, fresh air, generous diet, and perfect quietude for a time. Should there, however, have been an hereditary taint in the system, or some great moral excitement at such a period, the attack will most likely not be prevented.

The only incurable case of this form of insanity which has occurred in my own practice arose from these causes:—A lady who, it was afterwards recollected, had evinced great oddity of manner during the latter period of her first pregnancy, had suckled her infant for ten months, when, unfortunately, her husband was attacked by an acute disease, and fell a victim to it within two or three days. Great strangeness of manner immediately was again apparent in the patient. Her bodily strength was much reduced, hallucinations came over the mind, the principal one being that her husband was still alive in his grave; and violent mania, succeeded by melancholy, was the result. Although four years have now elapsed since this attack, no evident change for the better has taken place.

In another case which has come within my knowledge, the lady, (mother of several children,) within four months of her last confinement, had complained of an undefined dread of something about to happen to

* A. C., æt. 40, married eighteen years, and the mother of three children, was confined with her last child on June 22nd, 1846. She is of fair complexion, and much emaciated. About two months since, the first symptoms of mental derangement evinced themselves, by her talking incoherently on religious matters, and by her attempting suicide. As her debility was extreme, her infant was taken from her, more especially as she had taken a great aversion to it. Since this period, she has been alternately better and worse, but for the last three weeks has at times been quite violent and unmanageable, and shown strong dislike to her children, although naturally a very fond mother. I first saw her on February 28th, 1847, in the lunatic ward, when she replied sensibly to most of the questions put to her. Her children, however, she enumerated by name to a very large number, and this daily increased, I found on questioning her, on the same subject; it, in fact, became the test as to her sanity, as she frequently for hours together was quite calm, and showed no aberration of mind on other points. She is occasionally seized with fits of violent trembling, and then talks violently and incoherently, fancying herself on the point of dissolution, wishing to see her husband, but never expressing the same desire as to her children. As she presented every symptom of physical exhaustion, good nourishment, tonics, and opium were administered whilst staying with us, and I believe the same mode of treatment was adopted at Hoxton, to which asylum she was transmitted on March 12th. On one of my periodical visits to our patients at that establishment, I was pleased to observe that on asking the usual question as to her number of children, she laughed (evidently remembering her former answers), but now answered correctly. She was very soon after discharged quite cured, on September 27th last.

her, and though in a debilitated state, would persevere in suckling her infant. At this juncture, unfortunately, a sudden death occurred in her house, which much shocked and distressed her. An attack of mania, accompanied by great violence of manner, suddenly supervened, and during her illness she was not able to recognise her husband and child. By weaning immediately, giving tonics, nutritious diet, keeping the patient perfectly quiet, and not allowing her to see her husband or children, reason was perfectly restored within three months, the catamenial discharge having previously recurred.*

Like the other forms of insanity, there is frequently observed a difference as to the mode of its aggression; it may sometimes suddenly break out, without the slightest previous suspicion on the part of the friends; but more commonly, there is an unusual and gradually increasing disturbance in the balance of self-control over the mental powers, which is even appreciable by the patient herself, until all guidance is lost, restraint gives way, and insanity appears in its full form.

The more usual form in which this appears, when dependent upon over-nursing, is that of melancholia, but not always so. I have seen several cases in which it assumed either the form of mania simply, or what is more common, a combination of melancholia with paroxysms of mania.

A case of this description occurred in my own private practice a few years since. Symptoms of phthisis having evinced themselves a few months after parturition in a young married lady, it was thought expedient that the opinion of an eminent physician should likewise be taken on the case. Owing to the fright and mental emotion caused by the anticipated examination, the night was passed without sleep. The excitement continued after the consultation, and speedily merged into melancholia, interrupted by occasional paroxysms of violent mania. In a few weeks, the symptoms gave way, and the patient recovered from the mental attack; but died, after some months, of the pulmonary disease.

From such opportunities as I have had for forming an opinion on

* C. V., æt. 24, of a calm temperament and happy disposition, sallow complexion and black hair, was confined with her *first* child eight months since. Her health has been good, but she has had great anxiety, owing to her husband having embarked in business, and lost all his property within the last few months. On his reaching home three days since, he found her singing hymns, and talking to herself on religious subjects, and she has since that time been almost incessantly occupied in a similar manner. She was brought into the female lunatic ward of the infirmary on October 12. There is no violence in her manner, but she speaks almost continuously in a low, soft voice, quoting Scripture very correctly, and is evidently very conversant with it, and with the church service. This is varied occasionally by singing psalms and praying. She accuses herself of murder, of denying her Saviour, and of various other crimes, but has a placid expression of countenance, and does not appear unhappy. The tongue is white but not coated, pulse weak, skin cool, and her features pale and emaciated. Sleeps very little, but is not violent; perspires much. Bowels relieved properly, and urine likewise passes freely. There is a quiet, continuous lamentation in well-expressed language, and the case evidently is one proceeding from exhaustion combined with distress of mind, which has been inwardly preying on her, but which she has hitherto concealed from her husband, by his account. As the law is now imperative on the point, she was removed after a few days to the lunatic asylum at Bethnal-green, but it is a case which no doubt, with quiet and generous living, will ultimately do well.

the subject, it appears that insanity dependent upon over-nursing is more common amongst the poorer classes than in those who are provided with all the necessary comforts; for in the former, there is not only the drain upon the system by suckling, but this is often combined with a very insufficient supply of food, owing to their poverty, whilst, added to it, is the continued daily bodily exertion which is as constantly requisite, that they may exist. If I remember aright, however, this is contrary to the experience of Esquirol.

It was the opinion of Puzos, Levret, and some other writers on this subject, that a transition of milk to the brain was the cause of this form of the disease; but few, if any, medical practitioners of the present day will subscribe to this view. Esquirol mentions that nurses (especially among the poor) are more liable to suffer from this complaint *after weaning* than whilst nursing; but it is a question whether the fact of weaning itself was not in these cases found to be absolutely requisite, owing to the debilitated state of the system. Weaning *suddenly*, without any precautions or care, is stated on good authority to have sometimes brought on an attack of insanity. Esquirol mentions nineteen such cases as having occurred in his practice, and first appearing a few days after weaning.

It is a well known fact that some other great and continued debilitating causes, such as uterine hæmorrhage, for instance, acting on the system, will produce insanity, and several such cases are recorded.

It will not unlikely be found, however, on minute inquiry, that in all such cases as those now referred to, from whatever debilitating cause they may partly arise, that some moral shock or grief has also preceded the attack.

### PROPORTION OF CASES OF INSANITY ARISING FROM PUERPERAL CAUSES.

There is one point which requires explanation—viz., to account for the fact, that in the large lunatic hospitals a considerable proportion of the female cases admitted have arisen either after childbirth, or during suckling; and yet, on looking over the registers of extensive lying-in hospitals, it is surprising to find how rarely a case of insanity occurs in them: in the latter respect, the results of private practice will show the same result.

Thus, by *Esquirol's* statements, there were 1119 females admitted insane into the Salpêtrière at Paris during four years, of whom ninety-two were affected after childbirth; and in the higher classes of society the proportion was much larger, as in 144 cases, twenty-one had been thus affected either soon after childbirth, or during the period of suckling. The yearly admissions into the hospital of such cases is about one-twelfth of the total number received, and in some years one-tenth. In *Esquirol's* private practice, such cases form about one-seventh of the whole.

In the tables of 256 cases, of a higher order in society, admitted into *Charenton,* ten cases are recorded only as arising in consequence of parturition. (Annales d'Hygiène Publique, &c.)

*Dr. Haslam,* in a former report of cases in Bethlehem Lunatic Hospital, gives the proportion of puerperal ones as 84 in 1644.

Of the 899 females admitted into Bethlehem during the last five years, those labouring under puerperal insanity were 111—or 12·34; being one-eighth of the total number.

In the *Philadelphia* Lunatic Asylum, Dr. Rush has given the proportion as 5 in 70.

In the *Bethnal Green* Asylum, Mr. Phillips has been kind enough to examine the registers for the last three years, and finds, that out of 386 female patients, three only were the result of puerperal causes.

In the Grove House Asylum at *Bow*, Dr. Palmer, with the same kind feeling, has forwarded to me the results of 467 cases, of whom nineteen were thus affected, as seen in the accompanying table :—

| During Pregnancy. | Immediately after Delivery. | During Lactation. | Form of Disorders. | Results. | Average duration of treatment in the cases cured. |
|---|---|---|---|---|---|
| 1 | 6 | 12 | Mania . 15<br>Melancholia, 4 | Recovered . 14<br>Recovering . 2<br>Removed, probably recovered, 1<br>Recurrt. Mania, 1<br>Hopeless Dementia . . 1 | 4 months. |

Of the nineteen cases, three had had previous attacks of insanity unconnected with pregnancy, and two had before been affected by puerperal mania. In eight cases, an hereditary tendency to cerebral affections existed.

I have been unable to obtain any statement as to *St. Luke's*.

The following is an abstract which I have made from the reports of 703 female cases admitted into the *Hanwell Asylum*, within the last eight years :—

| | |
|---|---|
| Puerperal . . . . . . . . | 26 |
| In pregnancy . . . . . . . | 4 |
| Whilst nursing . . . . . . . | 7 |
| After miscarriage . . . . . . | 2 |
| Suppression of milk . . . . . | 6 |
| Hysteria . . . . . . . . . | 7 |
| Suppressed menstruation . . . . | 10 |
| Critical period . . . . . . . | 8 |
| Uterine excitement . . . . . . | 9 |
| Total . . | 79 |

Of 805 cases examined by Dr. Conolly in 1839, he found only one case of puerperal mania remaining there.

Again in 1845, on examining the female cases, there were *two* of puerperal mania only.

In the *York Retreat*, out of 246 cases, there were from

| | | |
|---|---|---|
| Puerperal disorders . . . . . | 9 | . . 11 |
| Undue lactation . . . . . . | 2 | |
| Irregular catamenia . . . . . | 10 | . . 13 |
| Hysteria . . . . . . . . | 3 | |

(Thurnam.)

I have thought it might, perhaps, be interesting to obtain a return of cases of insanity depending on the simple puerperal state, from some of the larger lying-in institutions of this metropolis, and the following is the result:—

In the *General Lying-in Hospital, Westminster*, in which the patients remain for three weeks after labour, out of the last 3500 cases delivered there, nine were affected with insanity. One apparently arose from a moral cause, after seeing her friends, but she recovered within four months: another was very slightly affected, and speedily was restored.*

In the *British Lying-in Hospital*, Dr. Henry Davies informs me that "within the last few years, there have been several cases of *modified* puerperal insanity, but no violent or acute ones; they have been despondent or melancholy, *and all recovered before leaving the hospital.*"

To Mr. Gream I am indebted for a detailed account of the statistics of 2000 cases, which have been delivered in the *Queen Charlotte's Lying-in Hospital*. Of this number there were eight cases of mania, and three combined with acute inflammation of the brain, excluding those cases in which delirium supervenes upon other diseases.

The number of insane cases here is larger in proportion than that of other similar institutions, but Mr. Gream accounts for it by the fact, that in this hospital the proportion of *unmarried* females is one-half of the total; many of these are already in a depressed condition, owing to anticipated destitution, and others become maniacal in consequence of the neglect of their friends or seducers.

Thus of the eleven cases, three were married, and eight unmarried.

I have confined myself to the returns of the in-patients of these hospitals, as, although the out-patients are more numerous, still there is less probability of arriving at a correct knowledge of them.

In the *Lying-in Wards of St. Giles's Infirmary*, amongst the last 950 cases, there was not one case of puerperal insanity, although a large proportion of the patients were single women; and of 1888 *out* cases, of which we there obtain immediate and accurate returns also, only *one* case of mania occurred, which was of short duration.

As to the usual age of patients thus attacked, the following table of M. Esquirol will, perhaps, afford the best answer.

In the 92 cases admitted into the Salpêtrière,—

| | | |
|---|---|---|
| 22 | were from . . . . . . | 20 to 25 |
| 41 | " . . . . . . | 25 to 30 |
| 16 | " . . . . . . | 30 to 35 |
| 11 | " . . . . . . | 35 to 40 |
| 2 | " . . . . . . | 43 |
| 92 | | |

Now, this result appears simply to accord with the ages of women relative to fecundity; and as the greatest proportion of these will be found from twenty to thirty, the cases of puerperal insanity will be somewhat in the ratio as to the proportional number confined at each period of life stated, or not far from it.

* Three were acute cases, and proved fatal; one from phrenitis and coma; another followed an attack of puerperal convulsions.

Thus, in one of the tables which I published formerly, and which contained the ages of the patients, the latter will be found in the following proportion in 1771 cases:—

| | |
|---|---|
| Age when confined—under 20 | 69 |
| Between 20 and 30 | 1100 |
| " 30 and 40 | 542 |
| " 40 and 45 | 54 |
| " 45 and 50 | 6 |
| Total | 1771 |

ON THE CAUSES OF PUERPERAL INSANITY.

In considering this subject, even as connected with general insanity, the fixing on any determinate physical cause is attended with the greatest difficulty. Various appearances have been described as presenting themselves, on post-mortem examination of the brains of those who have died whilst labouring under this disease; but independently of there being no peculiar and constant pathological lesions, we are also unable to decide whether the appearances which are described have been cause or effect.

There are, however, several causes combined in the pregnant and puerperal states, which may give rise to the disease now under consideration. It has already been shown that any unusual excitement or irritability of the uterine organs, when long continued, may *alone* produce symptoms of insanity: such, for instance, as suppression of the menstrual function; the act of conception; pregnancy; the suppressed or disordered lacteal secretion, or lochial discharges, which have all in turn occasionally furnished cases for the lunatic asylum. Sympathy of the brain with the disordered state of the functions of the uterus, especially if combined with those of the other abdominal organs, is here the apparent cause.

Those who are more particularly conversant with the treatment of female complaints, are well aware to what an extent disturbance of the cerebral organs will proceed from any derangement of the uterine functions, as evidenced by headache, giddiness, loss of memory, confusion of thought, throbbing in the temples, depression of spirits, &c. But when we find that, combined with the vast changes which occur in the uterine organs during pregnancy, and more rapidly immediately after parturition, there is superadded an acknowledged state of great nervous excitability, a change even in the physical characters of the circulating fluid, and above all, the influence of *moral* causes to so great an amount, (causes which, according to Pinel, Esquirol,* and others, much more frequently produce insanity than do the *physical*,) we can scarcely feel surprised at its aggression under such disadvantageous circumstances, but rather that it is not much more frequently observed.

The great exhaustion after labour, the anxiety as to suckling, sleepless nights caused by the infant, excitement often from friends injudiciously calling and conversing with the patient, followed by the

* Esquirol computes that cases of insanity originating from *moral* causes are as four to one of those which are produced by *physical.—Memoire*, 1818.

physical excitement usually attendant upon the establishment of the proper secretion of milk,—all conduce to place her in such a state, that her mind is more accessible to an attack of puerperal mania. A case, some time since, came under my notice, in which it was caused by an alarm resulting from the entrance of a sheriff's officer to seize upon the goods.

Puerperal mania appears to be the effect, not of inflammatory action, nor even of congestion of the brain, but to depend more upon intense *irritability*. The treatment, when successful, is sufficient alone to prove this fact, for it will be seen that depletion by bloodletting in these cases is very seldom advantageous, but generally extremely prejudicial; nutritious food is required in almost every case, and even stimulants judiciously applied; whilst the few cases which prove fatal in acute mania will usually be found to sink principally from exhaustion.

Puerperal insanity may, then, be regarded as originating from excessive irritability of the whole cerebro-spinal system, combined with great depression or exhaustion. By this may be explained the fact that so little assistance has been afforded us by pathological anatomy, as no alteration of structure in the brain would be naturally expected to follow simple derangement of the functions, and the greater number of cases which have afforded opportunities for examination have been chronic ones of some standing, in which paralysis or slow chronic inflammation of the membranes have at length been superinduced.

Guislain remarks on " a sanguineous orgasm or erethism,"* a sudden injection of the cerebral vessels, in common mania, which does not amount to inflammation, but seems closely allied to irritability.

The dissections by Pinel, Esquirol, Georget, Greding, Haslam, and many other competent authorities, lead to the conviction, that in both general and puerperal insanity, " nothing decisive can be obtained in reference to it from any variations of appearance that have been hitherto detected."

Esquirol " has found nothing particular to point out the seat of disease in the brain." In some cases, the cerebral vessels were found quite emptied, and this fact, in accordance with similar cases by Gooch and others, shows the usually anæmic state of such patients.

Organic lesions of the brain certainly do not appear to be a common *effect* even of puerperal mania, for, like convulsions under similar circumstances, when either attendant upon or following parturition with a fatal termination, few, if any, traces are found in the encephalon.

The same cause will occasionally produce both puerperal *convulsions* and *puerperal mania*. A case of this description was received into the female lunatic ward of St. Giles' Infirmary, about three years since. The patient, aged twenty-two, eight months advanced in pregnancy with her first child, was very much alarmed by a fire which broke out in a house opposite to her own. Convulsions attacked her, and Mr. Robarts, of Great Coram Street, who attended the case, thought it advisable to forward the labour as much as possible, by dilating the os uteri, and

* " Traité de l'Alienation Mentale."

perforating the cranium of the fœtus. Two or three convulsive attacks came on even after the labour was terminated, accompanied by great stupor, on recovering from which, mania evidenced itself fully. I was requested to visit the patient, and she was, after being removed to the infirmary for a few days, sent to a lunatic asylum, from which she returned completely cured in about four months from the time of the attack. She has since that time borne twins on two separate occasions, Mr. Robarts informs me, without any return of a similar attack.

Another point of connexion between the two complaints is, "that each of them is more liable to attack the female in her first accouchement than in after ones.

The similarity of condition in the nervous system, as existing between delirium tremens and puerperal mania, has already been adverted to, both exhibiting great exhaustion of vital power, with much excitability.

Amongst the lower class of females who apply for entrance at the expected period of accouchement into parochial infirmaries, a large proportion of them are addicted to the daily use of ardent spirits; and, from some inquiries which I have instituted during the last three years, I have found that the habitual consumption of *opium* amongst the same class is to a far greater extent than is generally supposed by the public. I have been surprised at discovering how universal the practice has become, and to what an extent in some cases this drug is taken by them with impunity, or rather, without immediate fatal effects. It is not at all an uncommon circumstance in the infirmary for the head nurse to discover under the pillows of the patients a phial of laudanum or a box of opium pills, secretly put there for daily use ; and several young girls even have stated, on being questioned, that independently of the use of spirituous liquors, they are in the habit of purchasing daily their pennyworth, or more, of laudanum as a dram, and that there are favourite druggists' shops, at which they get better measure than elsewhere. It might naturally be expected, that at the period of labour, with the removal of such accustomed stimulus, and its usual consequences, we should find cases of puerperal mania much more frequent in this class, but the tabular statement already given does not bear out the fact. I may be allowed, however, to state, that in the last eighteen years, during which period I have had to sign the certificates of all those who are received as insane into the infirmary, previously to their being transmitted to the various lunatic asylums in the county, I have frequently of late been struck with the increase in number of cases of general insanity, which is certainly disproportionate to the annual increase of population; the habitual use of opium amongst this class may perhaps explain the fact.

In the pure puerperal mania, the disturbance of the system arising from the sometimes sudden suppression of the lochial discharge and the full advent of the lacteal secretion, is one of the principal and immediate exciting causes of the complaint, and, at all events, places the cerebral organs in such a condition that any great moral shock, or previous hereditary tendency, has then full opportunity of producing derangement of function to a greater or less extent. In the comparatively few cases which have fallen under my own notice in private practice or in consultation, the patients had all been previously remarkable for their excitable temperament or eccentricity of manner. *Taking cold* soon after confinement is another cause, acting, as it does often, in arresting the secretion of milk, the lochial discharge, and producing the consequent fever; the re-establishment of these secretions in a regular healthy condition is often the commencement of the recovery.

Morbid irritation of the *gastric* and *hepatic* organs will likewise be intimately connected with the appearance of this complaint, whether as cause or effect is I think doubtful, but it is quite certain that on a copious evacuation of dark vitiated secretions from the bowels, in some cases, an immediate amendment has been observable. The sympathy between the healthy and disordered function of the liver, and the condition of the cerebral organs, is too well known to all practitioners to require comment.

That *moral causes* have a most powerful influence in the production of puerperal mania, is a fact believed by most authors; and it may be further asserted, that it is a much more frequent cause of bringing out the symptoms of it, in constitutions already predisposed, than the *physical.* This is perhaps shown by the much larger number of cases which occur in unmarried women than in the married, as is also the case in general insanity. Thus, in the HANWELL report of cases, of 415 females there were 122 married, 263 unmarried, and 30 widows. In SALPÊTRIÈRE, of 1726 cases admitted, 397 were married, 980 unmarried, and 291 widows.

In the *German* reports, the proportion was still larger, according to Dr. Jacobi in his Statistik—viz., in 835 females 599 were unmarried, 80 widows, and only 156 married.

As a further proof that the same fact holds good as to puerperal mania, I may instance the statistical returns of those large lying-in institutions of London which have been given in these pages.

In the Queen Charlotte's Hospital, the proportion of insane cases is much larger than in the other two alluded to, and this return is very justly supposed to depend on the fact, that one half of the patients *there* admitted are *single* women. Of the eleven cases, three were married, and eight unmarried. It not unfrequently happens that young females of a superior class, who are deserted by their friends, are obliged to apply for admission into such an institution, to hide their shame, and for want of means; and of five fatal cases out of the eleven, one only was married. Two were of the class just described, highly educated, of sensitive dispositions, and compelled by the cruel desertion of their seducers, and neglect of their friends, to seek for admission into a public hospital.

It was supposed formerly that all puerperal diseases were the effect of the suppressed secretion and metastasis of the milk: thus, there was the milk abscess, the œdema lactea, the mania lactea, &c. Levret, in fact, asserted, that after the latter disease proving fatal, he had found milk within the cranium; but this was no doubt simply an albuminous effusion.

In conclusion, we may divide the *causes* of this complaint into *predisposing* and *immediate.*

The *former* will include, 1. Previous attack; 2. Hereditary predisposition; 3. Great mental shocks, as by fright, &c.; 4. Great natural excitability.

The *immediate causes* may be checked perspiration, and sudden suppression of the lacteal or lochial discharge.

### PROGNOSIS OF PUERPERAL INSANITY.

There are two points to be considered under this head—1. *The duration of the malady;* 2. *Fatal termination.*

#### DURATION.

In cases of insanity which occur *during pregnancy,* it is not uncommon to find that a cure takes place before the end of the term, without a recurrence in the puerperal state: several such cases are in the registers of lunatic asylums, I find. Much care, however, is required after labour in these cases, and additional reasons exist for avoiding all probable causes of mental excitement, as there is a chance of recurrent mania again attacking the patient. Thus, in the daily journals of 1841, a case was mentioned as discharged from St. Luke's, cured as it was thought; but soon after, having received a fright as to the child, she again became subject to the complaint, and in one of the paroxysms she destroyed both her infant and herself.

If the cases occurring during pregnancy are principally dependent on *functional* derangement, they will in general cease when such function is again properly re-established.

In the Hanwell report of 1841, a case is given as occurring during pregnancy in a girl of eighteen, who did not recover until fifteen months after the attack. Sir W. Ellis, however, gives it as the result of his experience, that such cases "generally improved as the term of gestation drew nigh, and *all* entirely recovered a few weeks after delivery."—(Treatise on Insanity.)

"A patient received into Hanwell had become insane three months after pregnancy had commenced, and laboured under complete melancholia. She was confined about two months after her admission, and the pains of childbirth at once aroused all her dormant feelings. The child was still-born, but all the secretions coming on in their natural course, she quickly recovered."—(Ellis.)

Sir W. Ellis says also, "If the treatment be commenced in the early stage of the disease, and there is no hereditary predisposition or powerful moral cause to keep up diseased action in the brain, it is one of the most curable forms of insanity."—(P. 242, *op. cit.*)

With regard to the probable duration of insanity in *puerperal* cases, most authors are agreed that they are more likely to recover speedily than in the other forms of the disease, and that *permanent* insanity is very rare when it arises from such causes.

Denman has remarked, that "this disorder, in some instances, ceases in twenty-four hours; in others, it continues only for a few days; in some, a few weeks, and in others, for several months; but the instances of its continuing more than six months are very rare, and there is scarcely one to be found who did not ultimately recover, if there was no previous disposition."—(Vol. ii. p. 510.)

Burns gives a similar opinion:—" In some instances, the patient recovers in a few hours; in others, the mania remains for several weeks, or even some months; but I believe it never becomes permanent, nor does it prove fatal, unless dependent on phrenitis."—(Principles of Midwifery, p. 544.)

Dr. Gooch says—" Of the many patients about whom I have been consulted, I know only *two* who are still, after many years, disordered in mind, and of these, one had already been so before her marriage."—(*Op. cit.*)

Esquirol, too—" Les aliénations mentales à la suite des couches, guérissent *généralement*, s'il n'y a pas de predispositions trop énergiques !" but immediately weakens the assertion by adding—" Il en guérit plus de la moitie," and giving fifty-five cases only out of ninety-two as cured.

Haslam returns fifty recoveries out of eighty-four puerperal cases, and Burrows thirty-five in fifty-seven.

The experience of practitioners, as to their individual patients, appears to be much more favourable than would be proved by the returns of different asylums. In some of these, however, it must be remembered that the cases, previous to their admission, have been treated elsewhere, and sent on account of the obstinate continuance of the complaint.

The duration of the malady will always be influenced by the previous melancholic or excitable temperament of the patient, or by an hereditary tendency, and the prognosis, therefore, will be more unfavourable, if any near relative has already become insane, and more so, should the patient herself have been previously attacked. It may, too, I think, be laid down as a general rule, " that those cases of puerperal insanity which do not recover within six months from the time of the attack, are very likely to become tedious and unsatisfactory, though eventually curable. As a general axiom, cases of melancholia will be longer in recovering than those of mania.

The time elapsing before recovery must, of course, in each case, be uncertain, and will depend upon various circumstances. Thus, in the *Hanwell* report for 1840, a female aged twenty-two, attacked by mania after parturition, was ill for six months, whilst another patient, aged forty-four, with a similar attack, remained for eighteen months; a third case, aged twenty-eight, whose insanity originated from undue lactation, coupled with domestic unhappiness, had two separate attacks of mania, her illness lasting altogether for *three years*. In the report of 1841, however, there is a case mentioned, aged thirty-one, and returned as cured after six years and a half; whilst another, aged forty-two, arising from fright and child-birth, was cured after five years' illness.

In 111 cases admitted into Bethlehem during five years, ending Oct. 31, 1847—

| | |
|---|---|
| Discharged, cured | 69 |
| Removed, or not cured | 24 |
| Died | 5 |
| Still under treatment | 13 |
| | 111 |

Of the thirty-seven recoveries in Dr. Burrows' table, twenty-eight were cured within the first six months.

A fair opinion may be formed in future as to the duration of illness in pauper lunatics, as the parochial authorities are obliged, by a late act of parliament, to report any such casè to the magistrates within three days, and it is then immediately transferred to a lunatic asylum.

Formerly, "very few of the cases of puerperal insanity, after delivery, were brought to the asylum at Hanwell, until after the lapse of many weeks or months," according to Sir W. Ellis; and this admission of chronic cases will, perhaps, explain the unsatisfactory result, as obtained from the tables of some asylums, as to the proportion of cures effected.

The following is a tabular statement of the DURATION OF DISEASE in the sixty-nine puerperal patients cured in Bethlehem:—

| | | | |
|---|---|---|---|
| Under 2 months | 2 | Under 8 months | 7 |
| „ 3 „ | 4 | „ 9 „ | 4 |
| „ 4 „ | 15 | „ 12 „ | 3 |
| „ 5 „ | 6 | Above this | 7 |
| „ 6 „ | 9 | The original reports being defective, duration not ascertained in | 6 |
| „ 7 „ | 6 | | |

In the SALPETRIERE, of 55 cases cured—

| | |
|---|---|
| 4 | were in the 1st month. |
| 7 | „ 2nd „ |
| 6 | „ 3rd „ |
| 7 | „ 4th „ |
| 5 | „ 5th „ |
| 9 | „ 6th „ |
| 15 | in the following months. |
| 2 | after 2 years. |
| 55 | —or about two-thirds within the first 6 months. (Esquirol.) |

FATAL TERMINATION.

If we include the cases of *phrenitis* which have been already alluded to, as occasionally though rarely attacking the puerperal patient, the prognosis in such cases will be always most unfavourable. Their rare occurrence, however, is best shown by taking a table, such as that of Dr. Collins, in which, out of 16,414 cases delivered in the Dublin Lying-in Hospital, three only died of phrenitis.

In what is termed *acute mania*, it may perhaps be doubtful if there is not a subacute inflammatory action of the brain mixed up with the other common symptoms of mania. The complication of such a state, directly after childbirth, with depressed vital powers, not only must excite alarm in the mind of the medical practitioner, but renders the treatment exceedingly difficult in steering between the depletive and supporting remedies which may be here required. It is this description of case which Gooch described as so dangerous, with the quick pulse and febrile symptoms. In proportion as cases approximate to the form of phrenitis, so much the more dangerous do they become.

*Exhaustion* appears to be the principal source of danger; the want of sleep, intense excitement, and monotonous self-fatigue, all combine to increase it, and it is often a matter of surprise to us, for what an extent of time the human frame can withstand their effects.

Should even the mental symptoms somewhat improve, yet if the insomnia still continues, with a quick weak pulse, and other increasing symptoms of bodily debility, the termination of the case is to be looked to with apprehension.

In instances of *simple* puerperal mania or melancholia, the great majority may be considered as likely to recover eventually: many practitioners whom I have questioned on the subject have never met with a fatal case amongst their private patients, and I may state this as my own experience likewise. If we consider puerperal mania to be produced by functional derangement of the brain, and not from organic disease, it will explain the favourable anticipations as to the result of such cases.

More danger seems to be attached to cases of *single* women after confinement, when attacked by mania, than in the *married*, especially if their minds are susceptible of the grief, shame, remorse, effect of neglect, and destitution, so likely to overwhelm them at such a time. It will be remarked, that out of the five fatal cases already mentioned as occurring in a lying-in hospital, from mania, *one* only was married.

In the report of the 111 cases at Bethlehem, which Dr. Webster has forwarded to me, five only proved fatal, and these all had commenced within fourteen days after delivery—the earliest, four days after, the latest, fourteen. Thus, one commenced four days after first confinement, insane fifteen days; one five days after third confinement, insane three months; one eight days after first confinement, insane thirteen days; one ten days after sixth confinement, insane ten weeks; one fourteen days after seventh confinement, insane four months.

Of the ninety-two puerperal cases in Salpêtrière six proved fatal; one in six months after labour; one after one year; two after a year and a half; one after three years; one after five years.

Esquirol, in giving this statement, asks why it is that the *abdominal* affections after accouchement are so fatal in comparison with the *cerebral?* The question is easily answered. In the *former*, they depend upon *inflammatory* action pervading the serous coverings of a large surface, and that action, too, often of an erysipelatous typhoid character. In the *latter*, it is simply a derangement of function *when* simple mania; but when inflammatory action does attack the brain, *then* the mortality is as great as that caused by the abdominal disease.

In Dr. Burrows' table of fifty-seven cases, ten died, or about one in six.

From all the inquiries which I have been enabled to make on the different points connected with puerperal insanity, it would appear as a result, "that the great majority of such cases, whether occurring from pregnancy, parturition, or from undue lactation, recover, and that the mortality is comparatively very small."

#### LIABILITY TO AFTER ATTACKS.

This is by no means so great as we should at first be inclined to suspect; for although Esquirol mentions one case of mania after the first labour, in which the female was successively attacked in a similar manner after each of her eleven succeeding ones, each attack lasting about a month or six weeks; and likewise a second case in which mania appeared after *five* labours successively, yet numerous instances occur, in which no return takes place, though strong exciting causes may have existed, such as great misfortunes, domestic chagrins, &c. I have myself mentioned one, in which the patient bore twins twice, without any similar attack.

In speaking as to this liability on future similar occasions, Esquirol naïvely observes, "On prévient les accès, en évitant la grossesse."

The patients mentioned by Gooch had no return in their subsequent confinements, with one exception.

One of the Bethnal-green patients, already alluded to, was attacked after her *first* labour, recovered in three months, and although she has since had several other children, there has been no recurrence of the mania.

Still a judicious practitioner will, on a succeeding confinement, employ every precaution to prevent any undue excitement taking place. We now proceed to consider the treatment of puerperal insanity.

(*To be continued.*)

---

